# Pancreatic ectopic thyroid tissue: A case report and analysis of literature

**DOI:** 10.1515/biol-2022-0742

**Published:** 2023-10-24

**Authors:** Hai-lan Zheng, Xiao Lin, Ming-jian Zhang, Guo-hua Zhang

**Affiliations:** Department of Radiology, Taizhou First People’s Hospital, 218 Hengjie Road, Huangyan District, Taizhou City, HuangYan, 318020, Zhejiang Province, China; Department of Pathology, Taizhou First People’s Hospital, HuangYan, 318020, China

**Keywords:** ectopic thyroid, pancreas, abdominal computed tomography

## Abstract

Ectopic thyroid is a rare malformation induced by a migration defect in the developing gland during embryogenesis. In 90% of cases, the ectopic thyroid is located in the lingual region, whereas it is extremely rare in the abdominal cavity, particularly in the pancreas. A 50-year-old female patient presented to the Taizhou First People’s Hospital with a complaint of recurrent mid-lower abdominal pain and diarrhea for approximately a month. The abdominal computed tomography scan revealed a space-occupying lesion with abundant blood supply in the head of the pancreas during the consultation. This led to the suspicion of a neuroendocrine tumor. The doctor considered that this lesion in the head of the pancreas could be responsible for the patient’s incontinence. A laparoscopic pancreaticoduodenectomy was performed after relevant tests were undertaken and contraindications were ruled out. The patient was diagnosed with ectopic thyroid of the pancreas through postoperative pathology. Ectopic thyroid can be considered in middle-aged and elderly women who present with a mass with abundant blood supply and an unknown diagnosis. Subsequent treatments should be decided after fine-needle aspiration cytology.

## 1 Introduction

The development of the thyroid begins during the third or fourth week of gestation, commencing at the base of the pharynx between the first pharyngeal sacs. A rare malformation caused by a migration defect in the developing gland during embryogenesis, ectopic thyroid refers to the failure of the thyroid to descend from the thyroid anlage region to its final location in front of the trachea [1]. The prevalence of ectopic thyroid is between 1:100,000 and 1:300,000 in the healthy population and between 1:4,000 and 1:8,000 in patients with thyroid diseases. The gland can be found anywhere along the path of migration, with the lingual region being the most common location (90%) and the abdominal cavity being extremely rare [2]. Here, we report a case of imaging misdiagnosis ectopic thyroid of the pancreas and review the relevant literature.

## 2 Case presentation

The patient, a 50-year-old female, was admitted to Taizhou First People’s Hospital with a complaint of recurrent mid-lower abdominal pain accompanied by diarrhea for approximately a month. The patient had been experiencing unexplained mid-lower abdominal pain (relatively severe paroxysmal bearing-down pain without radiating pain, and no apparent correlation with eating), accompanied by diarrhea (yellow, pasty, and mucous stools, approximately ten times per day). In addition, abdominal pain was alleviated following defecation, accompanied by abdominal distension and poor appetite; absence of black, bloody, or bloody mucopurulent stools; absence of tenesmus, acid regurgitation, and belching; and no chills or fever. These symptoms were not significantly improved after self-administration of Changyanning tablet, levofloxacin, and gentamicin. Since the onset of the disease, the patient’s mental state had remained normal, despite diminished physical strength, a poor appetite, normal sleep, no significant changes in weight, defecation as described previously, and normal urination. The patient was in excellent health and had no personal or menstrual history to note.

The results of the physical examination were as follows: clear mind, normal mental status, no abnormalities in the heart and lungs, a flat and soft whole abdomen, tenderness in the mid-lower abdomen, no rebound tenderness, no palpable abdominal masses, no liver and spleen palpable below the costal margin, percussion pain in the hepatic and renal regions (negative), Murphy’s sign (negative), tenderness at McBurney’s point (negative), four times per minute bowel sounds, shifting dullness (negative), succession splash (negative), no edema in both lower extremities, and normal muscular tension in the extremities. Laboratory tests showed no significant abnormalities in blood routine parameters, ultrasensitive C-reactive protein, urine routine parameters, stool routine parameters, stool culture, liver and kidney function, or tumor markers.

**Figure 1:**
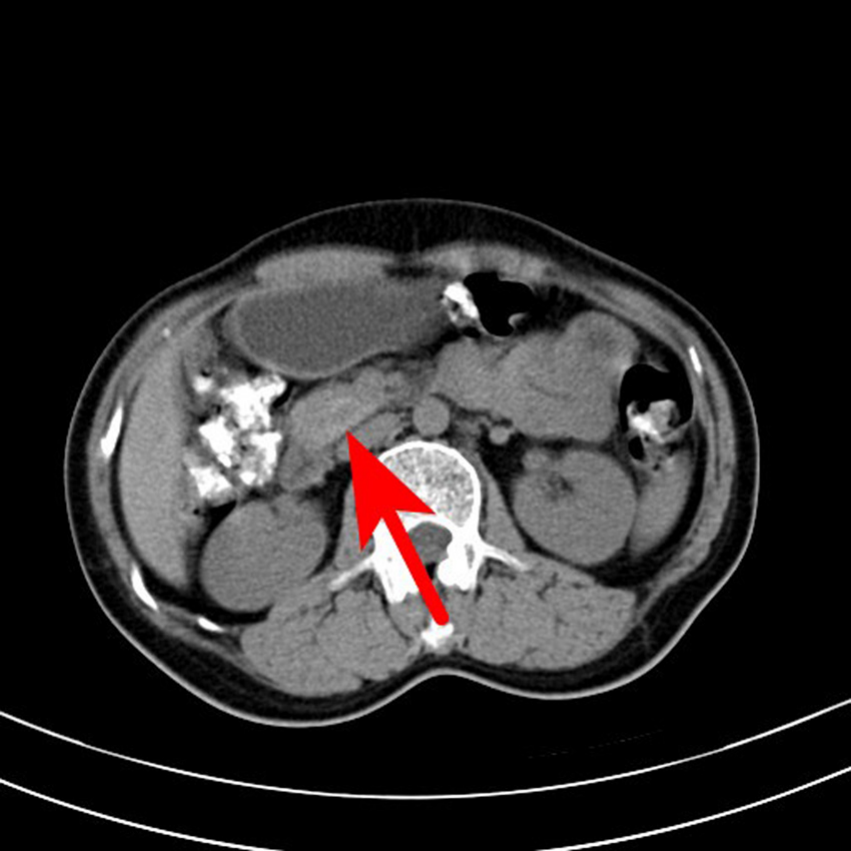
CT scan of the abdomen of the patient. The arrow points to a nodular slightly high-density shadow on the head of the pancreas, with a maximum transverse diameter of about 19 mm and a clear boundary, and a small saccular low-density shadow and a punctate calcification shadow.

**Figure 2:**
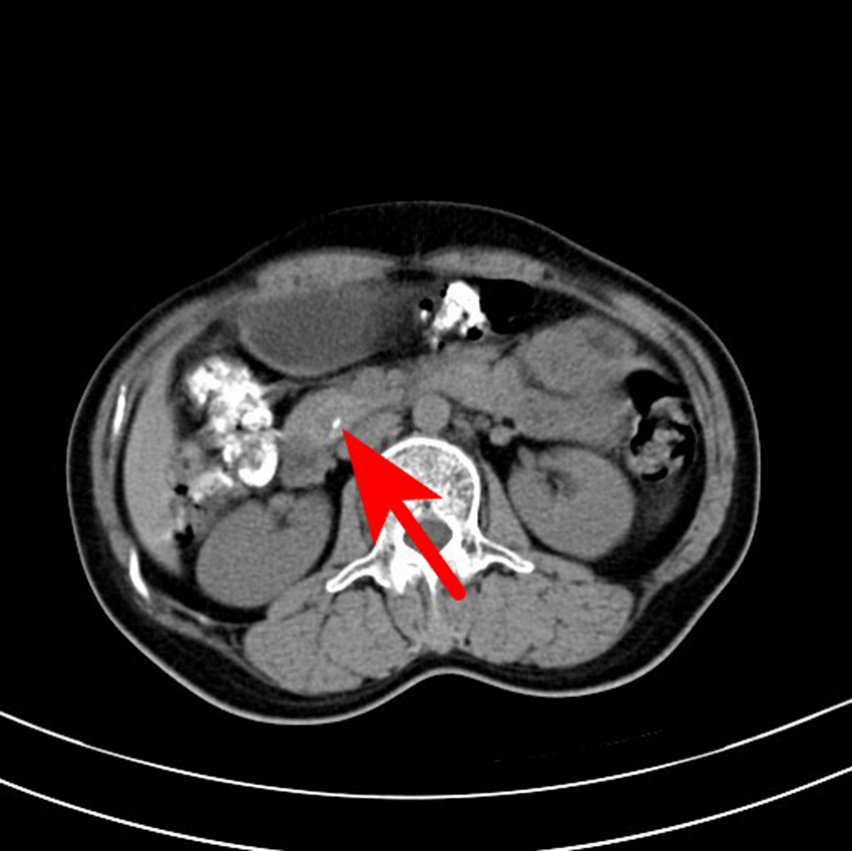
CT scan of the abdomen of the patient. The arrow points to a nodular slightly high-density shadow on the head of the pancreas, with a maximum transverse diameter of about 19 mm and a clear boundary, and a small saccular low-density shadow and a punctate calcification shadow.

**Figure 3:**
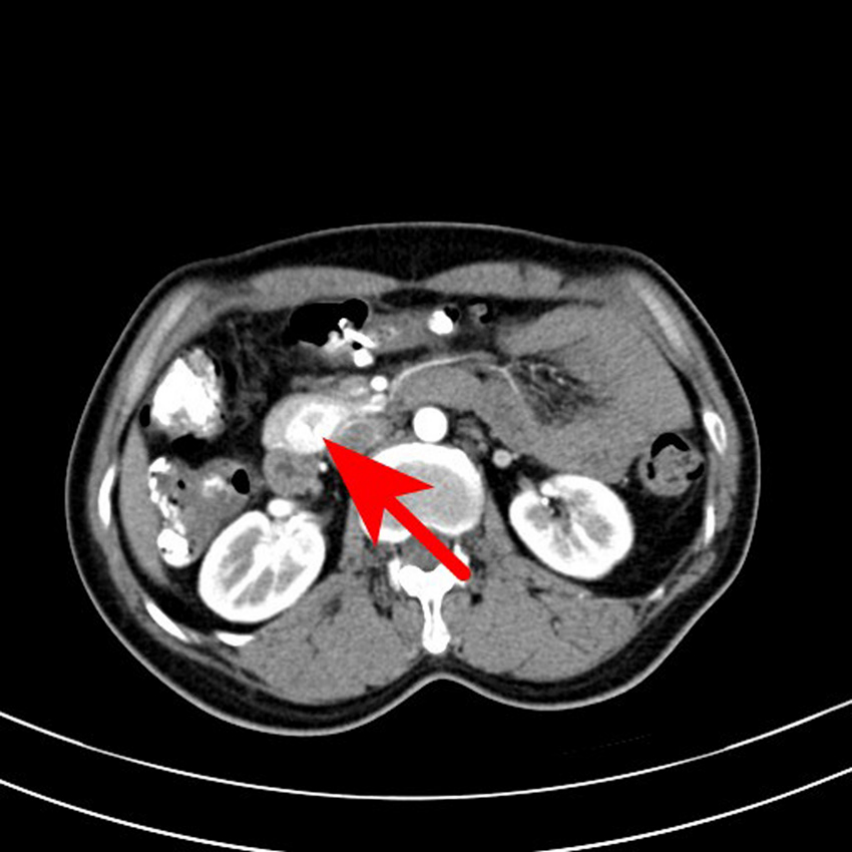
Transverse axial scan of the arterial phase. The lesions showed uneven enhancement, the highest CT value reached 192 HU, and the low-density areas were not enhanced.

**Figure 4:**
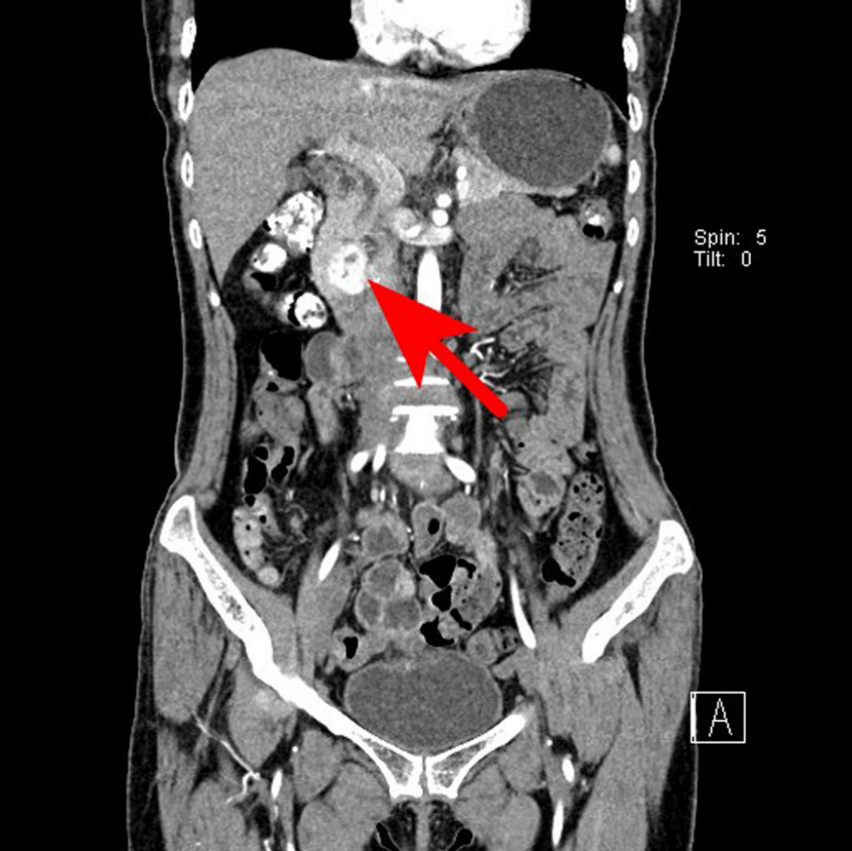
Coronal scan of the arterial phase. The lesions showed uneven enhancement, the highest CT value reached 192 HU, and the low-density areas were not enhanced.

**Figure 5:**
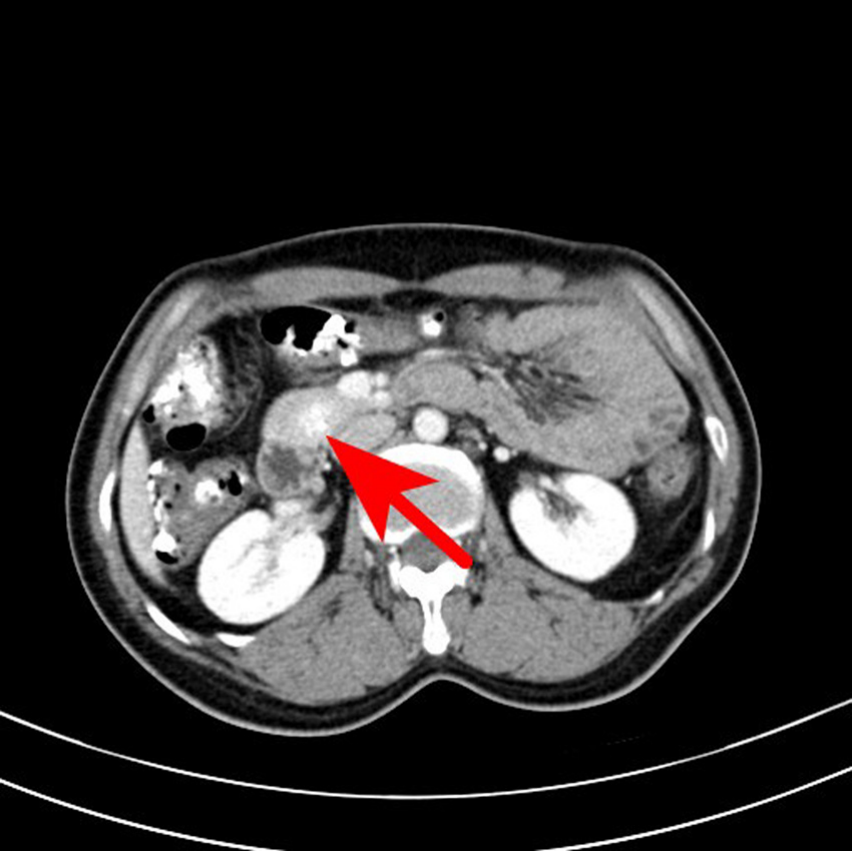
Transverse axial scan of the portal venous phase. The lesion was still high-density with CT value of 174 HU.

**Figure 6:**
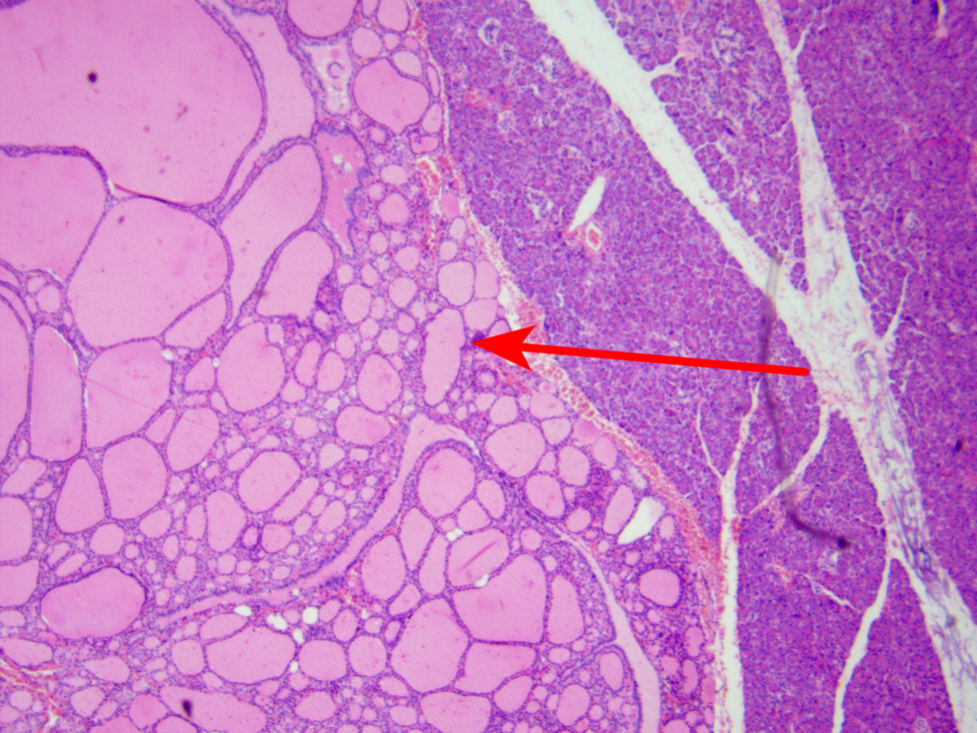
Pathological findings: HE staining, 40×; under the microscope, thyroid tissues were observed within the pancreatic tissues, approximately 2.5 cm × 2.0 cm × 1.6 cm in size, with follicles of varying sizes and mild cell morphology.

The following imaging tests were conducted. Specifically, the enhanced computed tomography (CT) of the entire abdomen revealed a slightly dense nodular shadow in the head of the pancreas with a maximum transverse diameter of approximately 19 mm, clear borders, and a small cystic hypodense shadow and punctate calcification shadow within, as well as an obviously unevenly enhanced lesion and no enhancement of the hypointense shadow. The CT value was approximately 58 HU on the conventional scan, 192 HU during the arterial phase, and 174 HU during the portal venous phase, and the hypodense shadow was not enhanced. A neuroendocrine tumor was considered (Figures 1–6).

Clinical diagnosis and treatment were as follows: considering the correlation between the recurrent abdominal pain and diarrhea and a space-occupying lesion in the head of the pancreas, the patient underwent laparoscopic pancreaticoduodenectomy following relevant examinations, and after contraindications to surgery were excluded. Postoperative pathological examination demonstrated follicle-like structures of variable size with mild cell morphology in the pancreas, based on which ectopic thyroid tissues were suspected (Figure 6). Immunohistochemistry results indicated Ki-67 (+) <5%, thyroglobulin (TG) (+), thyroid transcription factor-1 (TTF-1) (+), cytokeratin 19 (CK19) (−), cluster of differentiation 56 (CD56) (+), AE1/AE3 (+), CD10 (−), neuron specific enolase (NSE) (−), paired box gene-8 (Pax-8) (+), progesterone receptor (PR) (−), and β-Catenin (+). Postoperative thyroid function examination showed 0.39 ng/mL total triiodothyronine ↓, 4.18 μg/dL total thyroxine ↓, 0.1577 μIU/mL high-sensitive thyroid stimulating hormone ↓, 0.76 ng/dL free thyroxine (FT4), and 1.53 pg/mL free triiodothyronine. Ultrasound of the thyroid suggested an enlarged thyroid on the left side and bilateral thyroid nodules (TI-RADS 3). The patient was discharged after a diagnosis of hypothyroidism and prescribed with levothyroxine tablets (50 μg, oral administration, qm). The patient was followed up for 4 months after discharge. The patient’s postoperative abdominal incision had healed, and thyroid function and other symptoms were normal.


**Informed consent:** Informed consent has been obtained from all individuals included in this study.
**Ethical approval:** The research related to human use has been complied with all the relevant national regulations, institutional policies and in accordance with the tenets of the Helsinki Declaration, and has been approved by the Ethics Committee of Taizhou First People’s Hospital.

## 3 Discussion

The most prevalent form of thyroid dysplasia, accounting for 48–61% of cases [3], is ectopic thyroid, which is the presence of thyroid tissue outside the normal anterior cervical region between the second and fourth tracheal cartilages. Ectopic thyroid can be divided into two types. The first type is that there is thyroid in the normal anatomical position in front of the neck, and there is thyroid tissue in the abnormal position, which is formed by the normal decline of some thyroid primordial tissue, called partial ectopic thyroid, also known as parathyroid gland. The second type is the absence of thyroid tissue at the normal anatomical position before the neck, while the abnormal area contains thyroid tissue. This is caused by abnormal descent of all thyroid primordial tissues, known as completely ectopic thyroid, also known as vagal thyroid. The case in this report is classified as a partial ectopic thyroid.

Since ectopic thyroid tissues are typically caused by arrested migration or excessive descent of the thyroid diverticulum [4], they are most often observed in the thyroid descent pathway (Wölfler zone) [5]. Ectopic thyroid tissue in the abdomen is rare, which is far from the embryonic developmental pathway and therefore challenging to explain by abnormal migration. Cassol et al. [5] reported that ectopic thyroid in the gastrointestinal tract, liver, and pancreas can be explained by heterogeneous or chemotaxonomic phenomena, as these locations share a common embryological origin from the foregut endoderm with the thyroid [6].

Notably, little is known about the molecular mechanisms implicated in ectopic thyroid development and normal lifelong thyroid function in certain patients [7]. However, it was reported that mutations in regulatory genes expressed in the developing thyroid may be responsible for the occurrence of ectopic thyroid [3]. In addition, genetic research has shown that mutations in genes regulated by transcription factors, such as TTF-1 (Nkx2-1), FOXE1, and PAX-8, are essential for thyroid morphogenesis and differentiation and may cause thyroid hypoplasia as mutations in these genes are associated with abnormal migration of the thyroid [3].

An ectopic thyroid can occur in various parts of the body, mostly in the head and neck, potentially in the mediastinum, chest cavity, and female reproductive system, and very rarely in the abdominal cavity [2]. There are few clinical reports on ectopic thyroid tissues in the abdominal cavity. There have only been 35 cases of ectopic thyroid in the abdominal cavity and three cases in the pancreas in the previous 60 years, according to literature available in English [1,8,9], and only one case in the pancreas in Chinese literature. Specifically, Seelig first described in 1997, a 69-year-old female patient who visited a hospital after experiencing 6 months of persistent epigastric pain despite clinical and laboratory testing being within normal limits. Two other cases of epigastric pain in female patients aged 50 and 73 years were reported. A case was reported in Chinese literature of a 37-year-old female asymptomatic patient who was found to have a space-occupying lesion in the head of the pancreas on physical examinations and suspected of solid pseudopapillary tumor on imaging examinations, with no significant abnormalities in laboratory tests including routine blood, urine, and stool tests, liver function tests, biochemical tests, coagulation tests, and tumor indicators, which were pathologically confirmed after laparoscopic pancreaticoduodenectomy. Therefore, the current case is the fifth report, in which a 50-year-old female patient presented to the hospital with a month of recurrent mid-lower abdominal pain accompanied by diarrhea, and an enhanced CT examination of the abdomen revealed a neuroendocrine tumor in the head of the pancreas. After the completion of all essential investigations, a laparoscopic pancreaticoduodenectomy was performed, and postoperative pathology verified the ectopic thyroid of the pancreas.

Imaging methods such as high-resolution ultrasound, CT, magnetic resonance imaging (MRI), and nuclear imaging can all be used to diagnose an ectopic thyroid. Ultrasound with the color Doppler technique is excellent for showing blood vessels, with a high correct diagnosis rate for entire ectopic thyroids in the neck and a low diagnostic rate for partial ectopic thyroids. Furthermore, this method has no specific presentation for ectopic thyroids in other locations, which causes a high misdiagnosis rate. CT scans and MRI are significant imaging tools for the examination of patients with ectopic thyroids, and they are especially beneficial when ultrasound cannot identify the ectopic thyroid. On CT, the ectopic thyroid appears as a hyperdense mass with clear borders; ectopic tissues have an inherent iodine content, calcification in some patients, and heterogeneous enhancement on enhancement scans. On MRI images, the ectopic thyroid reveals higher signals on T1- and T2-weighted images compared to the surrounding muscle tissue. MRI scans use less radiation exposure but are more expensive and require longer imaging durations than CT scans. Furthermore, the thyroid radionuclide test, such as ^131^I or ^123^I, has a high diagnostic yield but fails to detect a non-functioning ectopic thyroid [10]. As a result, the diagnosis of ectopic thyroid can only be confirmed by a pathological diagnosis. Surgery or puncture examinations can be used to obtain pathological specimens. Fine-needle aspiration cytology (FNAC) is regarded as the most accurate diagnostic procedure, with more than 95% correct diagnosis rate. When an ectopic thyroid is not identified, FNAC is a very useful diagnostic tool that can assist in the diagnosis of ectopic thyroid tissue, especially before surgery, and help the surgeon decide on further radioisotope imaging to determine if the mass is the only functioning thyroid tissue [11].

Laboratory tests typically reveal no significant abnormalities in the thyroid function of patients with an ectopic thyroid, but all lesions of normal thyroid tissues, including goiter and carcinoma, can occur in the ectopic thyroid. Patients with an ectopic thyroid who are asymptomatic and have normal thyroid function usually do not require treatment, but they must be observed. In symptomatic patients, treatment is dependent on symptoms, gland size, thyroid function, and histological findings [12]. Patients with severe obstructive symptoms, hemorrhage, ulcers, or suspected malignancy should undergo surgical intervention [1]. Furthermore, it is essential to confirm the presence of a normally located thyroid prior to surgery in order to prevent hypothyroidism. Surgical techniques include open surgery, laparoscopic surgery, and the use of the da Vinci surgical system. Due to technological constraints, open surgery has been the norm; however, with the development of minimally invasive technology, laparoscopy is widely used in surgery for abdominal ectopic thyroid, and the use of the da Vinci surgical system for resection has been reported [13]. Complications primarily determine the prognosis of patients with an ectopic thyroid.

## 4 Conclusion

Despite the existence of a few reports, ectopic thyroid in the abdominal cavity, especially in the pancreas, remains a rare condition. For undiagnosed abdominal masses, FNAC is recommended to determine the subsequent treatment option [7]. In particular, middle-aged and elderly female patients with a space-occupying lesion and abundant blood supply in the pancreas should be evaluated for an ectopic thyroid.
